# Designing plant flavonoids: harnessing transcriptional regulation and enzyme variation to enhance yield and diversity

**DOI:** 10.3389/fpls.2023.1220062

**Published:** 2023-07-28

**Authors:** Lina Jiang, Yifei Gao, Leiqin Han, Wenxuan Zhang, Pengxiang Fan

**Affiliations:** ^1^ Department of Horticulture, Zijingang Campus, Zhejiang University, Hangzhou, China; ^2^ Key Laboratory of Horticultural Plants Growth and Development, Agricultural Ministry of China, Hangzhou, China

**Keywords:** flavonoid, plant synthetic biology, transcription factor, enzyme diversity, plant chassis

## Abstract

Plant synthetic biology has emerged as a powerful and promising approach to enhance the production of value-added metabolites in plants. Flavonoids, a class of plant secondary metabolites, offer numerous health benefits and have attracted attention for their potential use in plant-based products. However, achieving high yields of specific flavonoids remains challenging due to the complex and diverse metabolic pathways involved in their biosynthesis. In recent years, synthetic biology approaches leveraging transcription factors and enzyme diversity have demonstrated promise in enhancing flavonoid yields and expanding their production repertoire. This review delves into the latest research progress in flavonoid metabolic engineering, encompassing the identification and manipulation of transcription factors and enzymes involved in flavonoid biosynthesis, as well as the deployment of synthetic biology tools for designing metabolic pathways. This review underscores the importance of employing carefully-selected transcription factors to boost plant flavonoid production and harnessing enzyme promiscuity to broaden flavonoid diversity or streamline the biosynthetic steps required for effective metabolic engineering. By harnessing the power of synthetic biology and a deeper understanding of flavonoid biosynthesis, future researchers can potentially transform the landscape of plant-based product development across the food and beverage, pharmaceutical, and cosmetic industries, ultimately benefiting consumers worldwide.

## 1 Introduction

**Figure 1:**
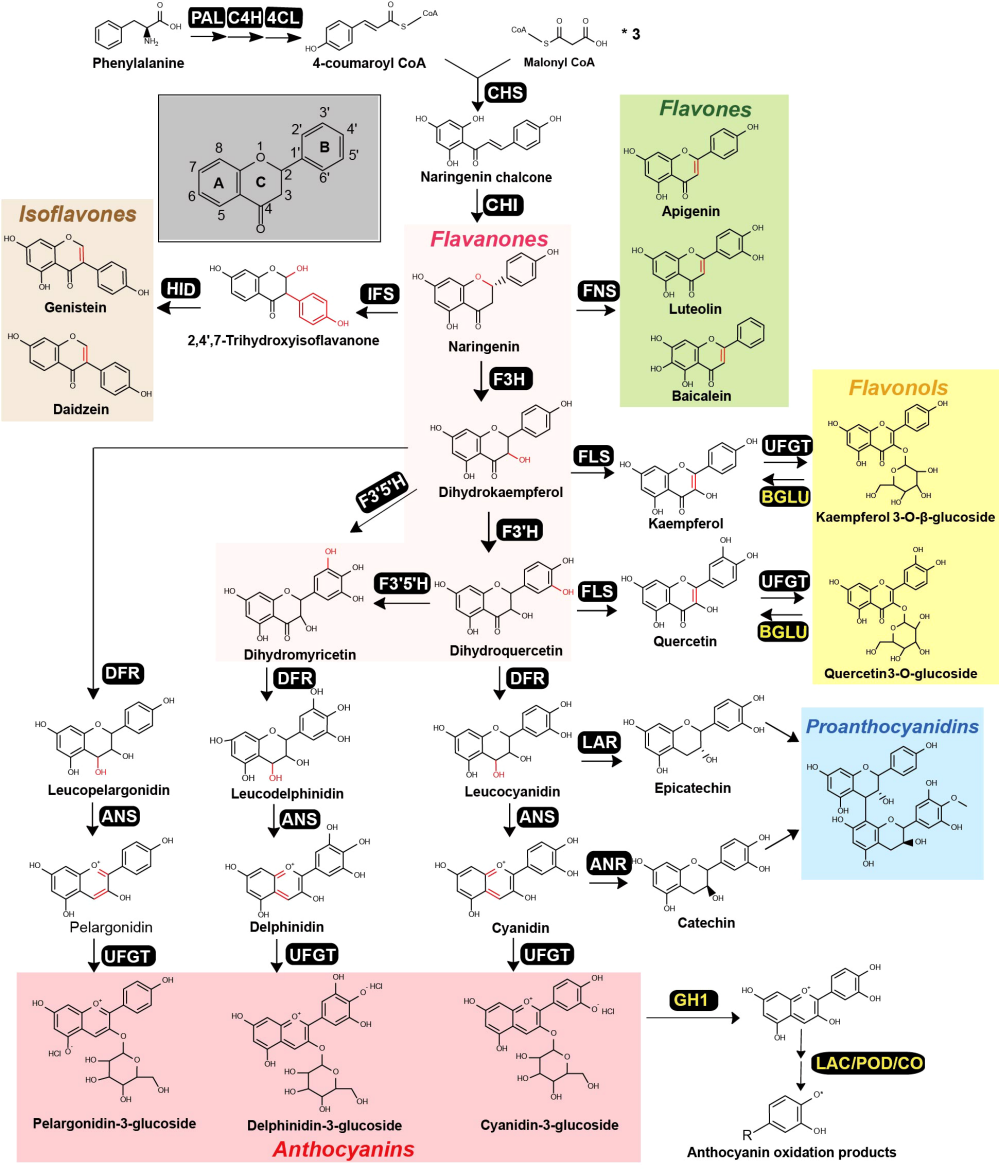
Canonical flavonoid metabolic pathway that produces a diverse range of flavonoids in plants. The pathway, beginning with phenylalanine, involves multiple enzymatic steps, yielding various flavonoid classes: flavanones (highlighted in pink), flavones (green), isoflavones (orange), flavonols (yellow), anthocyanins (red) and proanthocyanidins (blue). Each distinct enzymatic step is catalyzed by a specific enzyme, with abbreviation provided next to the corresponding product. These abbreviations include phenylalanine ammonia-lyase (PAL), cinnamate 4-hydroxylase (C4H), 4-coumaroyl-CoA ligase (4CL), chalcone synthase (CHS), chalcone isomerase (CHI), flavone synthase (FNS), isoflavone synthase (IFS), flavanone 3-hydroxylase (F3H), flavonoid 3’-hydroxylase (F3’H), flavonoid 3’, 5’-hydroxylase (F3’5’H), flavonol synthase (FLS), dihydroflavonol 4-reductase (DFR), anthocyanidin synthase (ANS), leucoanthocyanin reductase (LAR), anthocyanidin reductase (ANR), UDP-flavonoid glycosyltransferases (UFGT), *β*- glucosidase (BGLU), GH (glycoside hydrolase), Laccase (LAC), Peroxidases (POD), Catechol oxidases (CO).The chemical modifications mediated by each enzyme are highlighted in red within the respective chemical structures. The enzymes colored in yellow indicate that they are involved in flavonoid degradation steps.

Flavonoids are natural products derived from plants, characterized by a basic C6-C3-C6 structure formed by linking two benzene rings (A ring and B ring in **Figure 1**) via a heterocyclic pyrane ring (C ring). These compounds are widely distributed in plant tissues such as leaves, flowers, and fruits, and play a crucial role in plant-environment interactions. They protect plants from adverse environmental factors, such as UV radiation, and attract animals for plant pollination and seed dispersal (Grotewold, 2006; Falcone Ferreyra et al., 2012). Additionally, flavonoids have significant beneficial effects on human health (Winkel-Shirley, 2002; Grotewold, 2006; Falcone Ferreyra et al., 2012), acting as major bioactive secondary metabolites in medicinal herbs. They possess excellent free radical scavenging and antioxidant activities, reducing inflammation and the risk of chronic diseases such as cancer, diabetes, and cardiovascular diseases (Ravishankar et al., 2013; Lin et al., 2018; Perez-Vizcaino and Fraga, 2018; López, 2019). Diverse classes of flavonoids are currently manufactured and consumed in the food, pharmaceutical, and nutraceutical industries (Tian et al., 2017). However, the primary source of industrialized flavonoids relies on phytoextraction, which is constrained by limited supplies of raw plant materials containing the targeted compounds. In some cases, the desired flavonoids are present at low abundance in plants, further decreasing the product yields (Pyne et al., 2019; Sheng et al., 2020).

Modern biotechnology methods, featured by metabolic engineering and synthetic biology, are capable of introducing new biochemical pathways or rewiring existing metabolic networks in organisms to produce desired compounds. These techniques provide effective systems for developing biofortify plants with enhanced health-promoting chemicals (Martin, 2013; Lau et al., 2014; Fu et al., 2018). Historically, microorganisms such as *Escherichia coli* and yeast *Saccharomyces cerevisiae* have served as major hosts for metabolic engineering to produce the value-added natural products. For example, many flavonoids with nutraceutical and pharmaceutical interests have been produced by microorganisms, including flavanones (silybin and isosilybin, etc.) (Lv et al., 2019; Yang et al., 2020), flavones (luteolin and apigenin, etc.) (Pei et al., 2020; Zhu et al., 2020b), flavonols (quercetin and kaempferol, etc.) (Sultana and Anwar, 2008; Sheng et al., 2020). In recent years, plants have emerged as an alternative choice of synthetic biology chassis, particularly for producing plant-derived compounds. Although selecting plants as hosts poses challenges for metabolic engineering due to low transformation efficiency and long-life cycles, they offer several advantages, such as photosynthesis-driven growth using CO_2_ as the carbon source and the ability to expand production using existing agricultural or horticultural infrastructure (Zhu et al., 2017; Fu et al., 2018). Furthermore, plant chassis provides a promising platform to effectively generate plant-derive nutrients or bioactive chemicals that are difficult to produce in microbes, but can be more easily manipulated in plants by altering existing regulatory or biosynthetic enzymes (Zhu et al., 2017; Garg et al., 2018; Pyne et al., 2019).

Flavonoids are highly desirable compounds for plant metabolic engineering, and the flavonoid metabolic network has been successfully engineered in many plants to optimize the biosynthesis of health-promoting anthocyanins, resulting in anthocyanin-rich crops such as tomatoes, rice, and maize (Ogo et al., 2013; Zhang et al., 2015; Zhu et al., 2017; Liu et al., 2018a). Efforts to engineer desired flavonoids in plants have been pursued along two main directions: boosting flavonoid content by manipulating transcription regulatory components and introducing novel flavonoids through engineering enzyme diversity (Bovy et al., 2007; Nabavi et al., 2020). Simultaneously overexpressing multiple genes encoding biosynthetic enzymes efficiently promotes plant flavonoid contents, which can be achieved through manipulating master regulators, such as transcription factors (Hichri et al., 2011; Nabavi et al., 2020). Multiple transcription factors regulating flavonoids biosynthesis have been identified in various plant species, have been served as targets for plant flavonoid engineering. Concurrently, the natural variation of plant flavonoid biosynthetic enzymes, which exhibit promiscuous catalytic specificity, can be leveraged to expand chemical diversity and engineer flavonoids in selected plant hosts (Su et al., 2017; Jia et al., 2019; Shang and Huang, 2020; Jiang et al., 2021).

Overall, plant flavonoid metabolic engineering offers a valuable approach to modify the flavor, aroma, and color of plant-based products, ultimately enhancing their appeal to consumers (Zhu et al., 2020b). This can benefit the food and beverage industries, as well as for the pharmaceutical and cosmetic industries, where the demand for flavonoid-rich products is on the rise. In this review, we provide a comprehensive summary of recent research advancements in flavonoid metabolic engineering, encompassing the diverse benefits of flavonoids across various plant families, the transcription factors that govern flavonoid biosynthesis, and the enzyme promiscuity that can modulate flavonoid substrate specificity. Specifically, we emphasize the potential of utilizing transcription factors to increase plant flavonoid production and leveraging enzyme natural variation to expand flavonoid diversity as synthetic biology strategies.

## 2 Flavonoid biosynthesis and degradation

Flavonoids are a diverse group of secondary metabolites found in various plant families and can be categorized into several classes, including flavanones, flavones, isoflavones, flavonols, anthocyanins, and proanthocyanidins (Brodowska, 2017; Tohge et al., 2017; Dias et al., 2021). The canonical pathway for flavonoid biosynthesis begins with phenylalanine, which undergoes sequential catalysis by three enzymes, phenylalanine ammonia-lyase (PAL), cinnamate 4-hydroxylase (C4H), and 4-coumaroyl-CoA ligase (4CL), to produce 4-coumaroyl-CoA (Winkel-shirley, 2001; Falcone Ferreyra et al., 2012; Dias et al., 2021) (**Figure 1**). Chalcone synthase (CHS) then converts one molecule of 4-coumaroyl-CoA and three molecules of malonyl-CoA to naringenin chalcone, the essential precursor of flavonoids. Naringenin chalcone is subsequently converted to naringenin, a ternary ring structure, by the enzyme chalcone isomerase (CHI). Naringenin serves as a central metabolic node for various flavonoid classes (**Figure 1**), and can be converted to flavones (with a double bond between C-2 and C-3) by flavone synthase (FNS), to Fabaceae-specific isoflavones (with the benzene ring on C-3) by isoflavone synthase (IFS), or to dihydroflavonols (with C-3 hydroxylation) by flavanone 3-hydroxylase (F3H). Dihydroflavonols are further classified into dihydrokaempferol, dihydroquercetin and dihydromyricetin, depending on the position of B-ring hydroxylation at B-3’ or B-5’, which is catalyzed by flavonoid 3’-hydroxylase (F3’H) or flavonoid 3’, 5’-hydroxylase (F3’5’H) (**Figure 1**). Dihydroflavonols can also be converted to flavonols by flavonol synthase (FLS) similarly to FNS, forming a double bond between C-2 and C-3 positions. Furthermore, dihydroflavonols can be converted to anthocyanidins, a major group of flower pigments, by the sequential actions of dihydroflavonol 4-reductase (DFR) and anthocyanidin synthase (ANS). In some plants, leucocyanidin and cyanidin can be transformed to epicatechin by leucoanthocyanins reductase (LAR) and catechin by anthocyanidin reductase (ANR), respectively, which are further condensed to the polyphenol proanthocyanidins (**Figure 1**). Finally, UDP-flavonoid glycosyltransferases (UFGTs) catalyze the final glycosylation steps and are the major contributor to flavonoid chemical diversity.

Flavonoid catabolism is influenced by multiple factors including developmental signals and environmental conditions, including light, temperature, pH, etc. Current research exploring the catabolic enzymes responsible for flavonoid degradation mostly focusing on anthocyanins. These enzymes originate from various protein families including glycoside hydrolase (GH1) and oxidation related enzymes (LAC, POD, CO) (**Figure 1**). Enzymes in the glycoside hydrolase1 (GH1) family are primarily associated with the processing of plant defense-related glycosylated specialized metabolites, such as saponins, coniferin, flavonoids, and inactive glycosylated forms of plant hormones (Minic, 2008; Cao et al., 2017; Dong et al., 2019). Recent studies have found that GH1 plays a role in flavonoid catabolism, specifically targeting the β-glycoside linkage between glucose and the aglycone component of the flavonol glucosides (Bozzo and Unterlander, 2021). For instance, AtBGLU15 catalyzes the hydrolysis quercetin 3-*O*-*β*-glucoside to quercetin and glucose (Roepke and Bozzo, 2015; Roepke et al., 2017), while CsBGLU12 specializes in the hydrolysis of flavonol 3-*O*-*β*-glucosides, such as quercetin 3-*O*-*β*-glucoside (Baba et al., 2017). Laccases (LACs), copper-containing polyphenol oxidases, utilize oxygen to oxidize a broad spectrum of aromatic and non-aromatic compounds. Though primarily associated with lignin accumulation, certain LACs also contribute to the color fading of plant fruits, a process involving the breakdown of anthocyanins (Claus, 2004; Pourcel et al., 2007). Notably, a PbLAC4-like gene, identified in pears and regulated by the transcription factor PbMYB26, participates in anthocyanin degradation, causing a fading in the red hue of various pear tissues (Zhao et al., 2021b). In Arabidopsis seeds, the TT10 laccase in uniquely involved in flavonoid degradation through oxidation reactions among the 17 identified LACs (Pourcel et al., 2005). Flavonoid-peroxidases (PODs), primarily function as H_2_O_2_-scavengers, play a crucial role in plant cell detoxification (Yamasaki et al., 1997; Pourcel et al., 2007). In the case of *Brunfelsia calycina*, BcPrx01, a vacuolar type Class III peroxidase, was integral in the degradation of anthocyanins, accelerating the color transition of calyx flowers from deep purple to white after blooming (Zipor et al., 2015). In a separate study, Liu (Liu et al., 2022) observed an increase in POD activity coinciding with color fading, which is due to the anthocyanin degradation catalyzed by POD.

In summary, the multifaceted enzymatic machinery participating in flavonoid metabolism forms a complex metabolic network (**Figure 1**). This network serves as a blueprint for metabolic engineering strategies aimed at enhancing the production and modification of flavonoids in plants. By manipulating the key enzymes and regulatory factors within this system, researchers can unlock the potential for tailored production of specific flavonoids with desired properties, paving the way for the development of novel therapeutics, nutraceuticals, and functional food ingredients.

## 3 Chemical diversity, and potential health benefits of the flavonoid found across plant families

**Figure 2:**
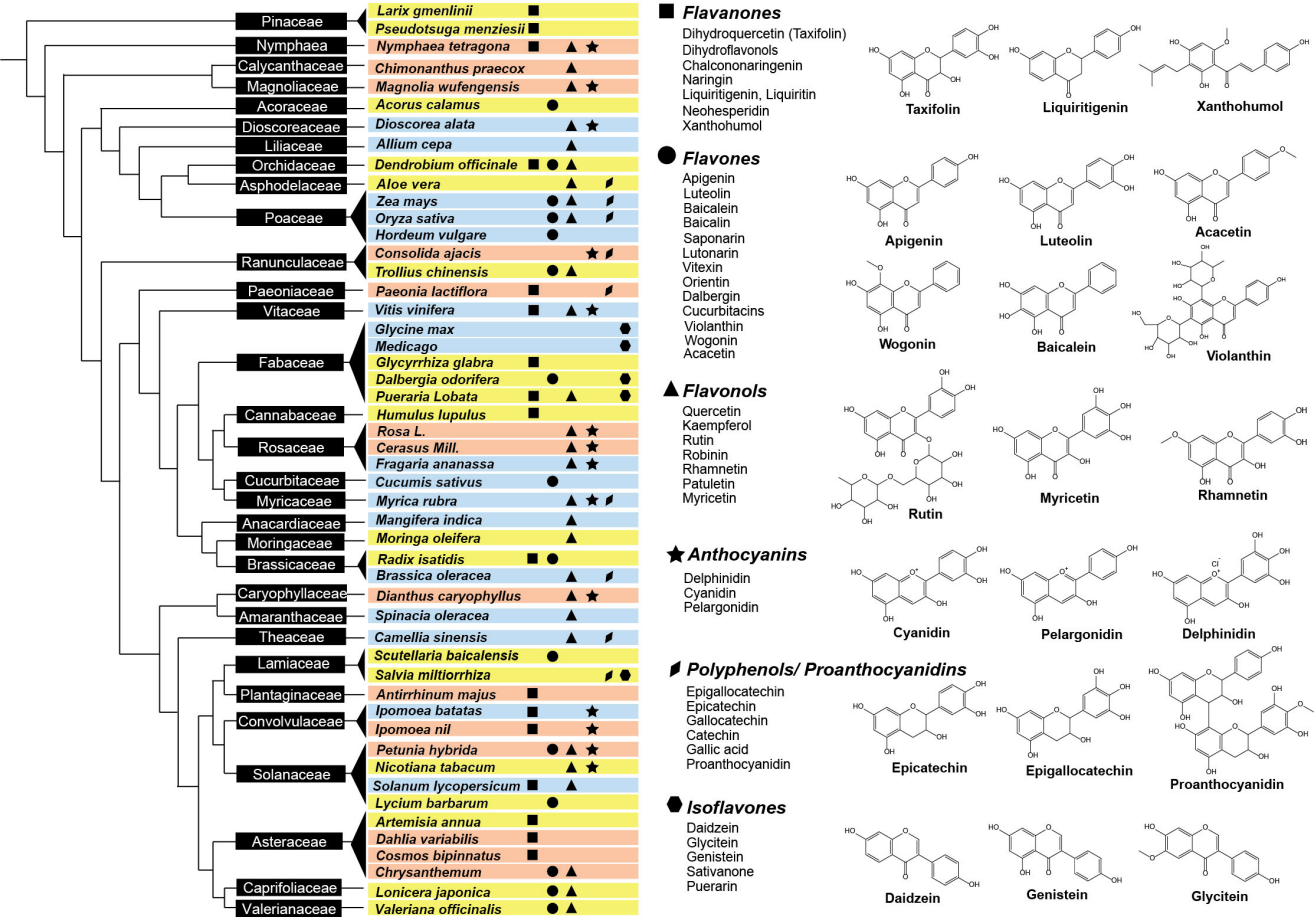
Distribution of flavonoid classes across plant phylogenetic tree. The left panel illustrates the phylogenetic distribution of representative plant species producing flavonoids with pharmaceutical (the corresponding plants are highlighted in yellow), nutritional (blue), or ornamental value (red). Plant families are displayed in black boxes. The right panel shows the major flavonoid classes produced by different plants, represented by unique shapes – squares for flavanones, circles for flavones, triangles for flavonols, stars for anthocyanins, diamonds for proanthocyanidins, and hexagons for isoflavones. Chemical structures of representative flavonoids from each class are also provided.

Flavonoids are widely distributed among different plant families and species, offering a range of benefits to humans (**Figure 2**). These flavonoid-rich plant species can be grouped into three categories based on their perceived benefits: medicinal plants with antibacterial or anti-inflammatory flavonoids, edible plants with health-promoting flavonoids, and ornamental plants with flavonoid pigments used for their floral coloration (**Figure 2**). Flavonoids have been identified as the primary bioactive compounds in various medicinal plants. These compounds have been extensively studied for their potential health benefits, demonstrating effectiveness in preventing and treating a variety of diseases. Baicalin and wogonin, found in the *Scutellaria* genus, have been shown to be effective in the prevention and treatment of cancer (Pei et al., 2022). Numerous studies have revealed their anti-tumor properties, inhibiting the growth and proliferation of cancer cells (Singh et al., 2021; Banik et al., 2022). Isoliquiritigenin and liquiritigenin, found mainly in the *Glycyrrhiza* genus, possess potent antioxidant and anti-aging properties. These compounds have been shown to protect against oxidative stress and inflammation, which are key factors in the development of aging-related diseases such as Alzheimer’s disease (AD) and Parkinson’s disease (PD) (Ramalingam et al., 2018). Silybin and isosilybin, discovered primarily in *Silybum marianum*, have a long history of use in traditional medicine for the treatment of liver disease. Modern research has confirmed the efficacy of these compounds in protecting the liver and promoting liver health (Lv et al., 2019). In addition to these specific compounds, other flavonoids, such as xanthohumol, apigenin, taxifolin, luteolin, and acacetin, have been found in various medicinal plants and have demonstrated diverse medicinal properties, including anti-inflammatory, antioxidant, and anti-cancer properties, among other health benefits (Tungmunnithum et al., 2018; Wang et al., 2018c; Ullah et al., 2020).

Plants have long been recognized as excellent sources of flavonoids, which not only aid in food preservation, but also promote a healthy diet (Sultana and Anwar, 2008). These compounds are commonly found in daily food and beverage consumption including fruits, vegetables, and tea, and have gained widespread attention due to their potent antioxidant properties – with some dietary flavonoids possessing stronger antioxidant effects even surpassing vitamin C and vitamin E (Sokół-Łętowska et al., 2007; Sultana and Anwar, 2008). Studies have shown that different plant species are abundant in diverse types of nutritional flavonoids. For example, soybeans are rich in isoflavones (Rochfort and Panozzo, 2007; Chen and Chen, 2021), yams are high in catechins (Dragovic-Uzelac et al., 2007), citrus fruits contain large amounts of flavones and flavanones (Dias et al., 2021), and red or purple fruits such as grapes, waxberries, and blueberries are abundant in anthocyanins (Sultana and Anwar, 2008; Terahara, 2015). Consuming these dietary flavonoids is not only beneficial for providing daily nutritional supplementation, but also helps regulate various body functions and maintain overall health (Yao et al., 2004; Sultana and Anwar, 2008; Terahara, 2015).

Flavonoids also play a role in adding color to various parts of plants, including flowers and leaves. Anthocyanin, one of the most well-known pigments, is estimated to contribute to the color of approximately 80% of flowers in angiosperms (Kong et al., 2003; Castañeda-Ovando et al., 2009). Chalcones and flavonols are primarily responsible for providing plants with a yellow color, while differences in the substituents present at various positions on the basic skeleton structure of flavonoids result in a diverse range of anthocyanins, generating colors such as red, purple, blue-purple, and blue (Castañeda-Ovando et al., 2009; Pasam et al., 2017). Flavonoids play a vital role in coloring various cut flower plants. For example, yellow color flower in common ornamental plants like rose, chrysanthemum, and hydrangeas are primarily due to the presence of chalcones, flavones, and flavonols, while orange, red, purple, and blue flowers, such as roses, tulips, commerinas, delphiniums, and violets are primarily due to the anthocyanin pigments (Nishihara and Nakatsuka, 2011; Iwashina, 2015; Iwashina and Mizuno, 2020; Stavenga et al., 2021). The so-called “black flowers”, such as *Fritillaria camtschatcensis* (Liliaceae) are also a result of the anthocyanins (Iwashina, 2003; Deguchi et al., 2016). It is worth noting that the role of flavonoids in plant coloration is not limited to visual appeal, but also contributes to attracting pollinators and providing protection against UV radiation.

The role of flavonoids in human daily life is multifaceted, and plants serve as a crucial and convenient source of these compounds. Although different plants contain varying amounts and compositions of flavonoid, with some plants generating limited types or producing low quantities overall, plant metabolic engineering has been used to increase the production of target components or broaden the chemical space of plant flavonoids (Pandey et al., 2016; Marsafari et al., 2020; Zhu et al., 2020b). By utilizing this approach, researchers have been able to enhance the content of existing flavonoids or create novel flavonoids not previously found in plants, providing a more efficient means to meet the nutritional and health needs of humans.

## 4 Transcriptional regulation of flavonoid biosynthesis pathway for metabolic engineering in plants

Plant flavonoid metabolism represents a complex and highly regulated process controlled by multiple transcription factors, which govern the gene expression of multiple steps within the flavonoid biosynthesis pathway. These transcriptional regulators can be harnessed for plant metabolic engineering, enabling the fine-tuning of gene expression to activate or repress metabolic pathway genes and enhance flavonoid content in plants (Broun, 2004; Grotewold, 2008; Hichri et al., 2011). In recent years, significant progress has been made in uncovering the biosynthesis-related transcription factors for flavonoids in numerous plant species as listed in **Table 1**. This offers promising opportunities for metabolic engineering to develop new and improved plant varieties with elevated flavonoid levels for human consumption.

**Table 1:** Recently identified transcription factors that regulate the expression of genes involved in plant flavonoid biosynthesis.

Plant Species	TF Name	Protein Family	Targeted Genes	Metabolites Regulated	*In vivo* experiment plants	Reference
*Actinidia chinensis* cvs	AcMYB123	MYB	*AcANS, AcF3GT1*	anthocyanin	*A. argute* (the fifth day) and Arabidopsis	(Wang et al., 2019b)
*Allium cepa*	MYB1	MYB	*-*	anthocyanin	Red onion	(Schwinn et al., 2016)
*Arabidopsis thaliana*	AtMYB12	MYB	*-*	genistein	Arabidopsis	(Pandey et al., 2014)
*Arabidopsis thaliana*	MYB111	MYB	*CHS, F3H, FLS1*	flavonol	Arabidopsis	(Li et al., 2019a)
*Arabidopsis thaliana*	MYB21	MYB	*FLS1*	flavonol	Arabidopsis	(Zhang et al., 2021)
*Camellia sinensis*	CsMYB2	MYB	*CsF3’H*	flavonol	–	(Wang et al., 2018d)
	CsMYB26	MYB	*CsLAR*	epigallocatechin		
Chrysanthemum	CmMYB012	MYB	*FNS*	flavone	‘Fencui’ chrysanthemum tissue cultures and early-flowering tobacco	(Zhou et al., 2021)
*Epimedium sagittatum*	EsMYB9	MYB	*EsCHS*	anthocyanin and flavonol	Tobacco	(Huang et al., 2017)
	EsMYBF1	MYB	*EsF3H, EsFLS*	flavonol		
*Epimedium sagittatum*	EsAN2	MYB	*-*	anthocyanin	Tobacco	(Huang et al., 2016)
*Fagopyrum esculentum*	FeMYBF1	MYB	*FLS*	flavonol	Arabidopsis	(Matsui et al., 2018)
*Lotus tenuis*	TT2b	MYB	*-*	proanthocyanidin	–	(Escaray et al., 2017)
*Lycopersicon esculentum*	SlMYB12	MYB	*-*	flavonol	3 tomato varieties	(Wang et al., 2018b)
Malus 3 domestic	MdMYB23	MYB	*MdANR*	proanthocyanidin	Apple calli and Arabidopsis	(An et al., 2018)
*Malus crabapple*	McMYB10	MYB	*McF3’H*	anthocyanin	Crabapple and tobacco	(Tian et al., 2015)
*Malus domestica*	MdMYB88	MYB	*MdUGT83L3*	anthocyanin	Apple callus	(Li et al., 2022)
*Malus sieversii* f. niedzwetzkyana	MYB22	MYB	*FLS*	flavonol	‘Orin’apple callus (yellow) and *Arabidopsis TT2* and *AtMYB12/-111/-11*	(Wang et al., 2017a)
	MYB12	MYB	*interact with bHLH3 and bHLH33*	leucoanthocyanidin		
*Medicago truncatula*	MtMYB134	MYB	*MtCHS2, MtFLS2*	flavonol	Arabidopsis and *M. truncatula*	(Naik et al., 2021)
*Medicago truncatula*	MtPAR	MYB	*act upstream of WD40-1.*	proanthocyanidin	–	(Verdier et al., 2012)
*Morella rubra*	MrMYB12	MYB	*MrFLS1*	flavonol	–	(Cao et al., 2021)
*Nicotiana tabacum*	NtMYB12	MYB	*NtCHS, NtPT2*	flavonol	Tobacco	(Song et al., 2020)
Peach	PpMYB9	MYB	*PpUGT78A2*	anthocyanin	Tobacco	(Zhou et al., 2016)
	PpMYB10.2	MYB	*PpUGT78B*	cyanidin		
	PpMYB17-20	MYB	*DFR*	repress anthocyanin		
Populus (poplar)	MYB182	MYB	*-*	anthocyanin and proanthocyanidin	Transgenic Poplar Plants	(Yoshida et al., 2015)
*Populus tomentosa*	MYB6	MYB	*DFR2, CCoAMOT1*	anthocyanin and proanthocyanidin	Arabidopsis and Leaf disks from *P. tomentosa* Carr.	(Wang et al., 2019a)
*Populus tomentosa*	PtrMYB57	MYB	*interact with bHLH131 and PtrTTG1*	anthocyanin and proanthocyanidin	*P. tomentosa*	(Wan et al., 2017)
*Populus tremula* 3 tremuloides	MYB165 and MYB194	MYB	*-*	repress flavonoids and phenylpropanoid	*P. tremula* 9 P. tremuloides (clone INRA 353–38) leaves	(Ma et al., 2018)
*Populus trichocarpa*	PtrMYB119	MYB	*PtrCHS1, PtrANS2*	anthocyanin	Arabidopsis	(Cho et al., 2016)
*Prunus persica*	PpMYB18	MYB		anthocyanin and proanthocyanidin	Tobacco leaf and Arabidopsis	(Zhou et al., 2019)
*Punica granatum*	PgMYB5-like	MYB	*F3H, F3’H, F3’5’H*	dihydroflavonols	*N. benthamiana*	(Arlotta et al., 2020)
‘Red Zaosu’ (*Pyrus pyrifolia* × *Pyrus communis*)	PpMYB17	MYB	*PpCHS, PpCHI, PpF3H, PpFLS, PpUFGT*	flavonol	Pear calli	(Premathilake et al., 2020)
*Rosa rugosa*	RrMYB5 and RrMYB10	MYB	*DFR, LAR, ANR*	anthocyanin or proanthocyanidin	Rose and tobacco	(Shen et al., 2019)
*Salvia miltiorrhiza*	SmMYB36	MYB	*-*	tanshinone, phenolic acid and flavonoids	Yeast strain AH109	(Ding et al., 2017)
*Schima superba*	SsMYB113	MYB	*SsCHS, SsNCED*	flavonoids	Arabidopsis	(Zhang et al., 2022)
*Solanum lycopersicum*	SlAN2-like	MYB	*SlMYBATVAC, SlAN1*	anthocyanin	Tomato cultivar AC	(Sun et al., 2020)
*Solanum tuberosum*	StMYBA1 and StMYB113	MYB	*DFR, F3’5′H*	anthocyanin	Tobacco	(Liu et al., 2016b)
Soybean	GmMYB100	MYB	*depress CHS, CHI*	isoflavonoid and flavone aglycones	–	(Yan et al., 2015)
Tomato cv zhongshu 4 (ZS4)	SlMYB14	MYB	*PAL*	flavonoids	3 tomato lines (OE2, OE5, and OE7).	(Li et al., 2021)
*Triticum aestivum*	TaMyb1D	MYB	*-*	repress lignin and flavonoids	Tobacco	(Wei et al., 2017)
*Vitis rotundifolia* Michx.	VrMybA1 and VrMYBCS1	MYB	*-*	anthocyanin	–	(Oglesby et al., 2016)
*Vitis vinifera*	VvMYBC2-L1	MYB	*-*	proanthocyanidin	Hairy roots of grapevine	(Huang et al., 2014)
*Vitis vinifera*	VvMYB5a	MYB	*-*	–	*P. hybrida*	(Cavallini et al., 2014)
	VvMYB5b	MYB	*DFR-A, ANS*	delphinidin		
	VvMYBA1	MYB	*VvUFGT*	petunidin and malvidin		
*Chimonanthus praecox*	CpbHLH1	bHLH	*AtPAP1, NtAN2*	anthocyanin	*Arabidopsis (Col-0)* and tobacco	(Zhao et al., 2020)
*Chrysanthemum morifolium*	CmbHLH2	bHLH	*CmCHS,CmDFR*	anthocyanin and proanthocyanidin	*Arabidopsis tt8-1* mutant	(Lim et al., 2021)
*Dendrobium candidum*	DcTT8	bHLH	*DcF3’H, DcUFGT*	anthocyanin	*Arabidopsis tt8* mutant	(Jia et al., 2021)
*Dracaena cambodiana* Pierre ex Gagnep	DcbHLH5	bHLH	*DcCHS1, DcCHS2, DcCHI1*	flavonoids	Tobacco	(Zhu et al., 2020a)
*Plagiochasma appendiculatum*	PabHLH	bHLH	*PaPAL, Pa4CL1, PaSTCS1, PaCHS, PaFNSI*	flavonoids	*P. appendiculatum* callus and thallus	(Wu et al., 2018)
*Plagiochasma appendiculatum*	PabHLH1	bHLH	*-*	flavonol and anthocyanin	*P. appendiculatum* thallus and Arabidopsis	(Zhao et al., 2019c)
*Selaginella lepidophylla*	SlbHLH	bHLH	*-*	total flavonoid	Arabidopsis	(Ariyarathne and Wone, 2022)
*Sorghum bicolor*	SbTT8	bHLH	*BAN (Banyuls)*	flavonoid PAs	*Arabidopsis tt8* mutant	(Salez et al., 2022)
*Camellia sinensis*	CsWD40	WD40	*-*	anthocyanin	Arabidopsis and tobacco	(Liu et al., 2018b)
*Medicago sativa*	WD40–1	WD40	*DFR*	anthocyanin	Alfalfa	(Feyissa et al., 2019)
*Malus domestica*	MdTTG1	WD40	*-*	anthocyanin	Apple callus tissue and Arabidopsis	(An et al., 2012)
*Solanum lycopersicum*	SlAN11	WD40	*SlFLS*	anthocyanin and proanthocyanidin	Tomato	(Gao et al., 2018)
*Artemisia annua*	AaYABBY5	YABBY	*AaPAL, AaCHS, AaCHI*, *AaUFGT*	anthocyanin and total flavonoid	*A. annua*	(Kayani et al., 2021)
Ougan (*Citrus reticulata* cv. Suavissima)	CitERF32, CitERF33 and CitRAV1	AP2/ERF	*CitCHIL1*	total flavonoid	Arabidopsis	(Zhao et al., 2021a)
*Solanum lycopersicum*	NF-Y	Nuclear factor Y	*CHS1*	naringenin chalcone	Tomato	(Wang et al., 2021)
*Vitis vinifera*	VvibZIPC22	bZIP	*VviCHI, VviFLS1*	kaempferol and quercetin	*N. tabacum*	(Malacarne et al., 2016)

### 4.1 R2R3-type MYB transcription factors and their roles in regulating flavonoid biosynthesis

**Figure 3:**
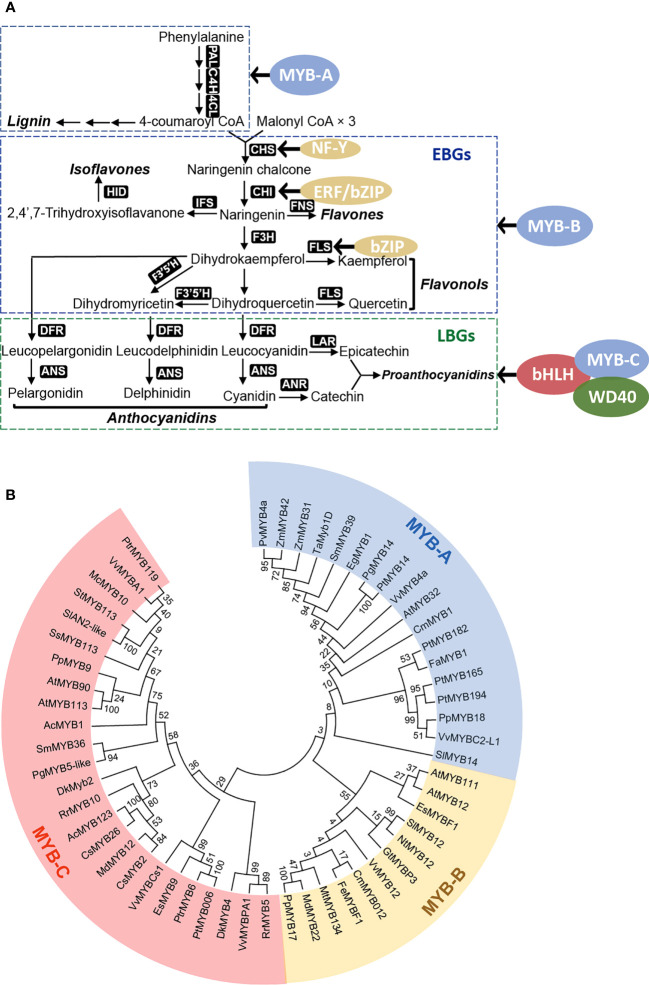
Transcriptional regulatory network in plant flavonoid biosynthesis. **(A)** Transcription factors critical for regulating genes in flavonoid biosynthesis, can be categorized into early biosynthetic genes (EBGs) and late biosynthetic genes (LBGs). Transcription factors belong to several protein families including MYB (blue), bHLH (red), WD40 (green), and other sporadically identified regulators (yellow); **(B)** Phylogenetic analysis reveals distinct groups of MYB proteins in regulating flavonoid biosynthesis. These recently identified R2R3-MYBs can been phylogenetically and functionally categorized into three groups: MYB-A (blue), MYB-B (yellow), and MYB-C (red), serving as the major regulators of flavonoid biosynthesis.

The regulation of flavonoid biosynthesis-related genes involves several well-characterized transcription factors, including the R2R3 MYB family, basic helix-loop-helix (bHLH) family, and WD40 proteins. These transcription factors are responsible for regulating the expression of genes in two primary groups (**Figure 3A**): the early biosynthetic genes (EBGs) and the late biosynthetic genes (LBGs) (Petroni and Tonelli, 2011; Falcone Ferreyra et al., 2012; Xu et al., 2015). EBGs, such as *CHS, FNS*, and *FLS*, encode enzymes involved in the biosynthesis of compounds upstream of dihydrokaempferol, including flavones and flavonols. LBGs, including *DFR, ANS, LAR*, and *ANR*, encode enzymes involved in the downstream pathway leading to the production of anthocyanins and proanthocyanidins (Dixon et al., 2005; Dixon and Sarnala, 2020) (**Figure 3A**). MYB proteins primarily regulate the EBGs, while LBGs are regulated by the MBW complex, comprising MYB, bHLH, and WD40 proteins (Li, 2014).

R2R3-type MYB transcription factors are the most frequently identified regulators of flavonoid biosynthesis. These factors bind to the cis-elements of promoters to regulate flavonoid metabolism (Dubos et al., 2010; Xu et al., 2015). Recently identified flavonoid biosynthesis-related R2R3-MYBs are phylogenetically divided into three groups that control the biosynthesis of specific flavonoid products (**Figure 3B**). The MYB-A group predominantly regulates genes involved in the upstream phenylpropanoid pathway and those associated with lignin biosynthesis. The MYB-B group mainly regulates the expression of EBGs, thus controlling the biosynthesis of flavonols or flavones, while the MYB-C group regulates the LBGs expression, determining the biosynthesis of anthocyanins or proanthocyanidins (**Figure 3B**). MYB-C group transcription factors often form a protein complex with bHLH and WD40 to execute their regulatory functions.

The MYB-B group, comprising MYB11, MYB12 and MYB111, is responsible for up-regulating the expression of essential EBGs, including *CHS, F3H* and *FLS1*. This leads to the accumulation of specific flavonols (Stracke et al., 2007; Stracke et al., 2010; Pandey et al., 2014; Pandey et al., 2015). AtMYB11, AtMYB12, AtMYB111 directly regulate the expression of *AtF3H* and *AtFLS*, impacting flavonol biosynthesis (Stracke et al., 2007; Li et al., 2019a). MYB12 has been extensively studied and primarily directs the flavonol biosynthesis. In tobacco, NtMYB12 regulates the expression of *NtCHS* and *NtPT2*, thereby increasing flavonol accumulation and enhancing tolerance to low phosphorus stress (Song et al., 2020). In the traditional medicinal plant S*cutellaria baicalensis*, the nuclear localized transcription factor SbMYB12 activates the expression of *SbCCL7-4*, *SbCH-2*, and *SbF6H-1* by binding to their promoters, resulting in increased baicalin and wogonoside content in the *SbMYB12*-overexpressed hairy roots of *S. baicalensis* (Wang et al., 2022). Ectopic overexpression of *MrMYB12* from *Morella rubra* in transgenic tobacco induced the expression of *NtCHS, NtF3H* and *NtFLS*, resulting in a significant increase in flavonol content and a pale pink or pure white flower color due to a decrease in anthocyanin content (Cao et al., 2021). Overexpression of *SlMYB12* in tomato fruit, using the fruit-specific *E8* promoter, significantly increased flavonol content (Wang et al., 2018b). In Asiatic hybrid lily flowers, an ornamental plant, *MYB12* upregulation under high temperature conditions enhances anthocyanin content and improves flower coloring (Yamagishi and Sakai, 2020; Yamagishi, 2022).

Several MYB variants in the MYB-B group, besides MYB11, MYB12, and MYB111, have also been identified to regulate the expression of EBGs in various plants. For instance, *MtMYB134* in *Medicago truncatula* enhances flavonol biosynthesis through the activation of *CHS* and *FLS* genes (Naik et al., 2021). The MYB17 transcription factor PpMYB17 from pear binds to the promoters of *PpCHS, PpCHI, PpF3H, PpFLS* and *PpUFGT*, increasing their expression and leading to higher levels of flavonols, flavanones, and flavones in *PpMYB17*-overexpressing pear calli (Premathilake et al., 2020). MYB22 in apple (*Malus sieversii*) stimulates the flavonol production by directly binding to the *FLS* promoter. In this case, *MdMYB22* was able to alleviate the flavonol deficiency phenotype in the Arabidopsis *AtMYB11/-12/-111* triple mutant, suggesting that MYB22 function is conserved with other MYB-B group members (Wang et al., 2017a).

The MYB-C group of transcription factors regulates flavonoid biosynthesis by forming a ternary protein complex with bHLH and WD40, referred to as the MBW complex. In *A. thaliana*, MYB-C proteins, such as MYB75, MYB90, and MYB113, along with bHLH family proteins TT8, GL3, and EGL3, have been identified as participants in leaf anthocyanin pigmentation (Borevitz et al., 2000; Gonzalez et al., 2008; Zuluaga et al., 2008). Only one WD40 protein, TTG1, has been identified in *A. thaliana* (Nesi et al., 2000; Zhang et al., 2003; Dubos et al., 2010), and it interacts with MYB and bHLH in various ways to jointly regulate anthocyanin biosynthesis (Xu et al., 2014; Xu et al., 2015). The same mechanism was also observed in the tea plant *Camellia sinensis*, where CsWD40 interacts with bHLH and MYB to form the MBW complex, regulating the biosynthesis of anthocyanin and proanthocyanidin (Liu et al., 2018b). In the edible plant mulberry, the transcriptional regulation of fruit flavonoids involves the cooperation of two MBW complexes, one regulating anthocyanin biosynthesis (MYBA-bHLH3-TTG1 complex) and another regulating proanthocyanidin biosynthesis (TT2L1/TT2L2-bHLH3/GL3-TTG1 complex). Both complexes activate the expression of MYB4, which fine-tunes the content of anthocyanins and proanthocyanidins in the plant (Li et al., 2020). In ornamental plants, the interaction between MYB-C group transcription factors and other components, such as bHLH and WD40, to form MBW complexes is frequently observed for the regulation of flavonoid biosynthesis. For instance, in coleus (*Solenostemon scutellarioides*), the MYB-C factor SsMYB3 forms an MBW complex with AtTT8 and AtTTG1 of Arabidopsis to regulate flavonoid biosynthesis (Zhu et al., 2015). Additionally, when SsMYB3 was expressed ectopically in tobacco, it interacted with two tobacco bHLH proteins (NtAn1a and NtJAF13-1) and a WD40 protein (NtAn11-1) to form an MBW complex that regulates the expression of anthocyanin-related genes *NtDFR*, *NtLAR*, and *NtANS* (Zhu et al., 2015). This suggests that MYB-C may be conserved across different plant lineages. Studies also showed that MYB-C group transcription factors may form complexes with only bHLH proteins, as not all plants contain WD40 proteins (Li et al., 2014; Xu et al., 2015). For example, in the medicinal plant *Epimedium sagittatum*, co-expression of *EsMYB9* with *EsTT8*, a bHLH gene, strongly induced the expression of LBGs, such as *DFR* and *ANS*, leading to increased anthocyanin accumulation (Huang et al., 2017; Jiang et al., 2020). Similarly, in the ornamental plant *Chrysanthemum* x *morifolium*, the interaction between *CmMYB6* and *CmbHLH2* enhances the expression of *CmDFR* and promotes anthocyanin accumulation (Lim et al., 2021).

On the other hand, some MBW complexes may also function as negative regulators in plant flavonoid biosynthesis, offering opportunities for fine-tuning flavonoid production in plant metabolic engineering. For example, in poplar trees, PtrMYB57 interacts with bHLH131 and PtrTTG1 to form an MBW complex that binds to the promoters of flavonoid genes, inhibiting gene expression and negatively impacting anthocyanin and proanthocyanidin biosynthesis (Wan et al., 2017). Similarly, in peach (*Prunus persica*), PpMYB18 interacts with PpbHLH3 and PpbHLH33 to inhibit the production of anthocyanin glycosides (Zhou et al., 2019). In the ornamental plant Japanese gentian (*Gentiana triflora*), GtMYB1R1 interacts with GtbHLH1 protein to form a GtMYB3-GtbHLH1 complex that controls floral anthocyanin biosynthesis. However, expressing *GtMYB1R* heterologously in tobacco flowers decreases the expression of *CHI, DFR* and *ANS* and reduces anthocyanin pigmentation (Nakatsuka et al., 2013).

In some instances, MYB transcription factors in the MYB-C group can regulate anthocyanidin biosynthesis even without a well-defined partnership with bHLH or WD40 proteins. For example, in peach, PpMYB9 or PpMYB10 have been found to bind to the promoters of various UFGT genes in petal flowers, influencing anthocyanin accumulation and, ultimately, petal color (Zhou et al., 2016). Similarly, in crabapple, McMYB10 has been shown to bind to the promoter of *McF3*’*H* and increase its expression, leading to enhanced anthocyanin accumulation in stably transformed crabapple plants over-expressing *McMYB10* (Tian et al., 2015). In grapes, the accumulation of anthocyanin pigments is regulated by a number of MYB-R2R3 transcription factors such as VvMYBC2-L1, VvMYB5a, VvMYB5b, and VvMYBA1. However, their relationships with bHLH and WD40 proteins have yet to be fully understood (Cavallini et al., 2014; Huang et al., 2014).

### 4.2 Regulation of flavonoid biosynthesis by bHLH and WD40 proteins of the MYB-bHLH-WD40 complex

The bHLH and WD40 proteins, integral components of the MYB-bHLH-WD40 (MBW) complex, play pivotal roles in regulating flavonoid biosynthesis in plants. The bHLH transcription factor family, encompassing various growth, developmental, and environmental adaptation processes in plants, has been found to be instrumental in the regulation of flavonoid biosynthesis (Samira et al., 2018; Zhu et al., 2019; Zhu et al., 2020a). Numerous studies have demonstrated the potential of leveraging bHLH proteins in modulating plant flavonoid production. For example, DcTT8, a bHLH protein in *Dendrobium candidum*, promotes the expression of *F3*’*H* and *UFGT*, leading to increased plant anthocyanin accumulation (Jia et al., 2021). Similarly, the bHLH protein DcbHLH5 from *Dracaena cambodiana* activates the expression of *CHS1*, *CHS2*, and *CHI1*, key genes involved in flavonoid biosynthesis, resulting in increased leaf flavonoid content and enhanced UV-B resistance (Zhu et al., 2020a). In the liverworts plant *Plagiochasma appendiculatum*, PabHLH1 positively regulates flavonol and anthocyanin biosynthesis by activating multiple genes involved in flavonoid biosynthesis, such as *PAL, CHS, CHI, F3H, DFR*, and *FLS*, leading to increased flavonoid and anthocyanin accumulation (Zhao et al., 2019c). PabHLH1’s functions appear evolutionarily conserved, as its heterologous expression in Arabidopsis also promotes the expression of EBGs and LBGs involved in flavonoid biosynthesis and results in increased flavonoid and anthocyanin accumulation (Wu et al., 2018; Zhao et al., 2019c). In another example, the sorghum bHLH transcription factor SbTT8 regulates the expression of genes related to proanthocyanidin biosynthesis. Heterologous overexpression of *SbTT8* in the Arabidopsis *tt8* mutant rescues the abnormal phenotype of seed dormancy and seed coat color by reestablishing proanthocyanidin biosynthesis (Salez et al., 2022).

WD40 proteins, composed of four or more highly conserved amino acid repeating units terminated by a tryptophan-aspartic acid (WD) dipeptide, are a widespread protein family in eukaryotes, participating in various biological processes such as signal transduction, transcriptional regulation, and cell cycle regulation (Neer et al., 1994; Stirnimann et al., 2010; Gao et al., 2018). In plants, WD40 proteins involved in anthocyanin biosynthesis typically form an MBW complex with bHLHs or MYBs to perform their functions (Xu et al., 2015; Lloyd et al., 2017). However, manipulation of the WD40 protein alone can also effectively control plant flavonoid biosynthesis. For instance, overexpression of the FtWD40 protein from *Fagopyrum tataricum* in tobacco enhances the expression of *DFR* and *ANS*, resulting in increased anthocyanin accumulation and pigmentation in petals (Yao et al., 2017). Similarly, overexpression of the tomato WD40 gene *SlAN11* in tobacco results in higher leaf anthocyanin and seed proanthocyanidin contents, while reducing flavonol accumulation as dihydroflavonol precursors are redirected to anthocyanin biosynthesis (Gao et al., 2018). In the ornamental plant *Freesia hybrida*, FhTTG1 interacts with FhbHLH proteins (FhTT8L and FhGL3L) to form the MBW complex. When FhbHLH is co-expressed with MYB and bHLH partners, significant activation of anthocyanin and proanthocyanidin biosynthesis is observed (Shan et al., 2019). Furthermore, WD40 proteins can regulate flavonoid biosynthesis through interaction with bHLH proteins, without the involvement of MYB. For example, the apple WD40 protein MdTTG1 interacts with bHLH but not MYB proteins to regulate anthocyanin biosynthesis (An et al., 2012). Similarly, SlAN11, a tomato WD40 protein, interacts with bHLH proteins but not MYB to promote leaf anthocyanin and seed proanthocyanidin accumulation, while reducing flavonol content (Gao et al., 2018).

### 4.3 The regulatory network of flavonoid biosynthesis beyond MYB, bHLH, and WD40 proteins

In addition to MYB, bHLH, and WD40 proteins, other transcription factors also play a role in regulating flavonoid biosynthesis. For example, a bZIP transcription factor, VvibZIPC22, was identified in grape to activate the promoters of flavonoid pathway genes (*VviCHI* and *VviFLS1*), leading to increased flavonol production (Malacarne et al., 2016). Ectopic overexpression of *VvibZIPC22* in tobacco promotes the expression of several floral genes involved in flavonoid biosynthesis, resulting in a significant increase in flavonoid content in the flowers (Malacarne et al., 2016). YABBY transcription factors, exclusive to seed plants, also play crucial roles in regulating primary and secondary metabolism, including flavonoid biosynthesis. In *Artemisia annua*, the *YABBY5* transcription factor activates multiple genes of the flavonoid pathway, including *AaPAL*, *AaCHS*, *AaCHI*, and *AaUFGT*, which led to a significant increase in total flavonoid accumulation and an increase in anthocyanin production that causes the stem to turn deep purple (Kayani et al., 2021). Nuclear factor Y (NF-Y) proteins, evolutionarily conserved transcription factors that bind to the CAAT box of many genes, have also been found to play a role in flavonoid regulation. In tomato, an NF-Y factor binds to the *CHS1* promoter and inhibits its expression, leading to a decrease in overall flavonoid content that typically imparts a yellowish color to the fruit during ripening, resulting in the production of pink-colored fruit with colorless peels (Wang et al., 2021). In citrus, the AP2/ERF transcription factors affect flavonoid biosynthesis. CitERF32 and CitERF33, two citrus AP2/ERF proteins, enhance the expression of *CitCHIL1*, enabling the substrate to flow more efficiently into CHS and thereby increasing overall flavonoid content (Zhao et al., 2021a).

In summary, transcription factors play a vital role in the regulation of flavonoid biosynthesis in plants, and their use in plant metabolic engineering holds great potential. By expressing selected transcription factors in specific organs, it is possible to manipulate the expression of key target genes involved in flavonoid production and thus control the levels of specific flavonoid components. This approach opens up a promising avenue to create plants with elevated flavonoid content, which are increasingly in demand for their health-promoting properties. However, it is important to note that while transcription factors are effective regulators of flavonoid biosynthesis, they mainly regulate certain classes of flavonoids. To specifically regulate the synthesis of a particular flavonoid, it is necessary to perform plant metabolic engineering experiments in combination with key structural genes.

## 5 Explore the natural diversity of flavonoid biosynthesis for synthetic biology

Plant-derived natural products have historically served as therapeutic agents, with their pharmacological properties largely determined by their core structures. However, to develop innovative drugs, it is often necessary to modify these structures (Guo, 2017). Such innovations can improve drug metabolic stability, increase activity or selectivity, adjust physicochemical properties, or simplify structures for easier production (Guo, 2017; Atanasov et al., 2021). The natural diversity of plant chemicals offers a reservoir of structural variations that can be exploited for drug innovation through metabolic engineering and synthetic biology (King et al., 2016; Lautié et al., 2020). Flavonoids are a class of plant natural products with potential for drug innovation, which are widely distributed throughout the plant kingdom and exhibit various structural modifications on their central structures, such as hydroxylation, methylation, and glycosylation. These modifications can significantly affect the bioactivity and bioavailability of flavonoid compounds used in drugs or functional foods. For example, methylation of the hydroxyl group in flavonoids can drastically improve their metabolic stability and membrane permeability, facilitating absorption and bioavailability (Wen and Walle, 2006; Koirala, 2016). Glycosylation of flavonoids can improve drug solubility and enhance uptake (Fernández et al., 2000). Metabolic engineering of flavonoid biosynthesis offers an opportunity to exploit the natural chemical diversity of flavonoids for drug innovation. By identifying and manipulating enzyme variants that can catalyze desired structural modifications, biosynthetic pathways can be simplified and enzyme performance improved. Such synthetic biology strategies hold great promise for developing new drug candidates and functional food ingredients, and for enabling more efficient and sustainable production processes.

### 5.1 Enzyme divergence at early flavonoid biosynthetic steps determines B-ring hydroxylation and methylation patterns

**Figure 4:**
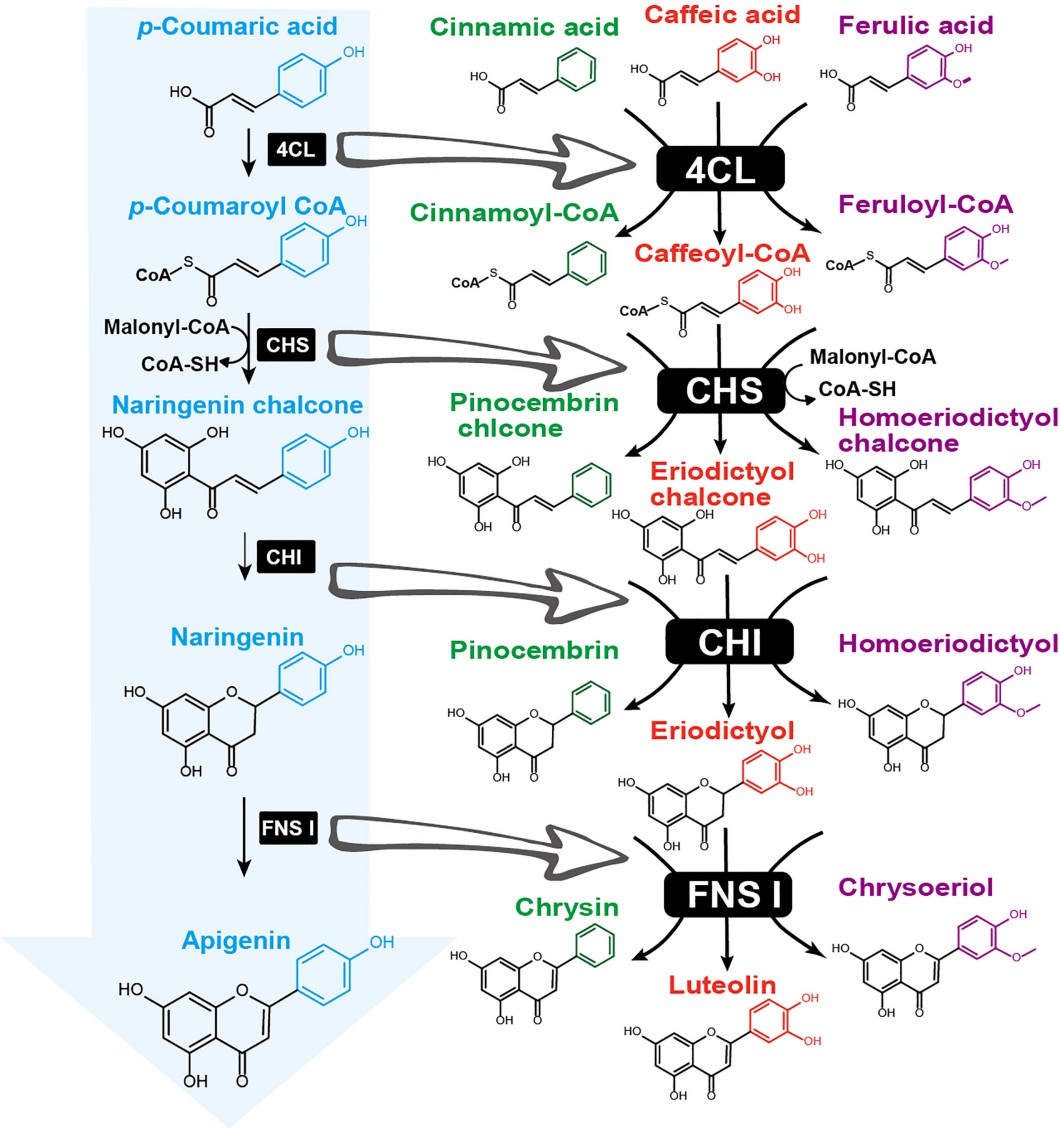
Enzyme promiscuity diversifies flavonoid B-ring hydroxylation and methylation patterns during early biosynthetic steps. The canonical flavonoid biosynthetic pathway (indicated by a blue arrow) begins with p-coumaric acid, which is sequentially catalyzed by 4-coumaroyl-CoA ligase (4CL), chalcone synthase (CHS), chalcone isomerase (CHI), and flavone synthase I (FNS I) to produce apigenin. Enzyme divergence led to promiscuous 4CL accepting multiple substrates with varying hydroxylation and methylation patterns on the aromatic ring (highlighted with different colors), including cinnamic acid (green), caffeic acid (red), and ferulic acid (purple). Acyl-CoA products are further converted by promiscuous CHS, CHI, and FNS to generate diverse flavones, such as chrysin, luteolin, and chrysoeriol.

Naturally, the flavonoid core structure is hydroxylated and *O*-methylated at various positions. The site and number of these substitution groups vary across plant species and have diverse physiological relevance, particularly in terms of flavonoid B-ring modification patterns. For example, six anthocyanidins, including cyanidin, pelargonidin, delphinidin, peonidin, petunidin, and malvidin, are differentiated by B-ring modifications and exist widely in flowers with colors of pink, scarlet, red, or magenta. The colors of these anthocyanidins are determined by the number of hydroxylations - a single hydroxylation on the 4’ position results in scarlet, while multiple hydroxylations on the 3’, 4’, and 5’ position give blue (Iwashina, 2015). In an extreme case, the *Scutellaria* genus of the Lamiaceae family produces unique 4’-deoxyflavones that lack any decoration on the B-ring, such as wogonin, baicalein, and chrysin, in addition to the 4’-hydroxyflavones apigenin and scutellarein (**Figure 4**) (Zhao et al., 2019a; Pei et al., 2022). Medical research has revealed that the 4’-deoxyflavones extracted from the roots of the Chinese herb *S. baicalensis* exhibit higher pharmacological activities than typical 4’-hydroxyflavones (Himeji et al., 2007; Zhao et al., 2019b).

The functional divergence of multiple enzymes in the early steps of flavonoid biosynthesis influences flavonoid B-ring diversity. In the canonical flavonoid biosynthetic pathway, the B-ring 4’-hydroxyl group is derived from *p*-coumaroyl-CoA, which is condensed with three malonyl-CoAs to produce the backbone naringenin chalcone that is catalyzed by CHS (**Figure 1**). The 3’- and 5’-hydroxylation are then generated by F3’Hs and F3’5’Hs. However, the 4’-deoxyflavones are less likely to be synthesized by the canonical pathway, which requires an as-yet unidentified dehydroxylase to remove the B-ring 4’-hydroxyl. Recent studies on *S. baicalensis* flavonoid biosynthesis have shown that enzyme divergence of *p*-coumaroyl-CoA ligase (4CL), CHS, and flavone synthase II (FNS II) leads to the production of unique 4’-deoxyflavones in the roots (Zhao et al., 2016; Zhao et al., 2019a). The divergence is initiated by a cinnamate-CoA-like enzyme, which prefers to activate cinnamic acid to yield cinnamoyl-CoA, in contrast to 4CL that generates *p*-coumaroyl-CoA using p-coumaric acid (Zhao et al., 2016). Then, a root-specific CHS2, separated from CHS1 after the divergence of the Lamiaceae family, evolved the function to produce pinocembrin chalcone, a flavanone precursor without B ring hydroxylation (Zhao et al., 2016). A regular CHI catalyzes pinocembrin chalcone to form pinocembrin, which is further converted to chrysin – the precursor of all other 4’-deoxyflavones – by a recently evolved, root-specific FNS II-2 (Zhao et al., 2016). Chrysin is then decorated on the A-ring by F6H to yield baicalein or by F8H and *O*-methyltransferase (PFOMT) to yield wogonin (Zhao et al., 2018; Zhao et al., 2019a). In the above case, the expanded substrate specificity of 4CL to activate cinnamic acid and CHS2 to accept cinnamate-CoA leads to the unique non-decorated flavonoid B-ring. In angiosperms, CHSs often undergo gene duplication and are expressed in different tissues or cell types with conserved biochemical functions (Huang et al., 2004; Zavala and Opazo, 2015). A study on the flavonoid biosynthetic pathway of the fern plant *Stenoloma chusanum* identified promiscuous 4CL and CHS that incorporate *p*-coumaroyl, cinnamoyl, caffeoyl, and feruloyl groups into flavonoids (Ni et al., 2022). The enzymes Sc4CL1 and Sc4CL2 can activate cinnamic acid and its three derivatives, *p*-coumaric acid, caffeic acid, and ferulic acid, to form the CoA-thioesters (Ni et al., 2022). ScCHS1 showed extraordinary substrate promiscuity towards these acyl-CoAs, which are condensed with malonyl-CoA to produce pinocembrin chalcone, naringenin chalcone, eriodictyol chalcone, and homoeriodictyol chalcone (Ni et al., 2022) (**Figure 4**).

In canonical flavonoid biosynthesis, hydroxylases and *O*-methyltransferases are required for decorating all positions on the B-ring except for the 4’ position (**Figure 1**). However, promiscuous 4CL and CHS enzymes can shorten the biosynthetic routes and generate flavonoid C6-C3-C6 core structures with predetermined B-ring decorations directly from phenylpropanoic acid. This is especially useful for metabolic engineering of flavonoids with desired B-ring modifications in microorganisms such as *Escherichia coli* and *Saccharomyces cerevisiae*. By feeding appropriate phenylpropanoic acids such as ferulic acid, engineered *E. coli* harboring *4CL1* and *CHS1* can efficiently produce homoeriodictyol (Cui et al., 2019; Dunstan et al., 2020; Ni et al., 2022), circumventing the need for hydroxylases and *O*-methyltransferases. When two additional genes, CHI and FNS I, are introduced, a diverse group of flavanones and flavones can be produced, further demonstrating the power of leveraging enzyme diversity for flavonoid metabolic engineering with synthetic biology (Li et al., 2019b; Ni et al., 2022).

### 5.2 Bifunctional enzymes in flavonoid biosynthesis: potential applications in metabolic engineering

Bifunctional enzymes are rare yet intriguing multifunctional proteins that catalyze two distinct biochemical reactions, often part of consecutive steps in a metabolic pathway (Moore, 2004). These enzymes have evolved through gene fusion, neofunctionalization following gene duplication, or retention of ancestral enzymes’ promiscuous activity (Moore, 2004; Copley, 2015; Seelig, 2017; Siddiq et al., 2017). The development of bifunctional enzymes was likely driven by the need for enhanced metabolic catalytic efficiency and improved coordination of biosynthetic steps. Consequently, bifunctional enzymes hold considerable potential for synthetic biology applications, simplifying biosynthetic steps and generating desired compounds more efficiently. In this section, we present two examples of bifunctional enzymes involved in flavonol and proanthocyanidin biosynthesis and discuss their potential applications in metabolic engineering.

**Figure 5:**
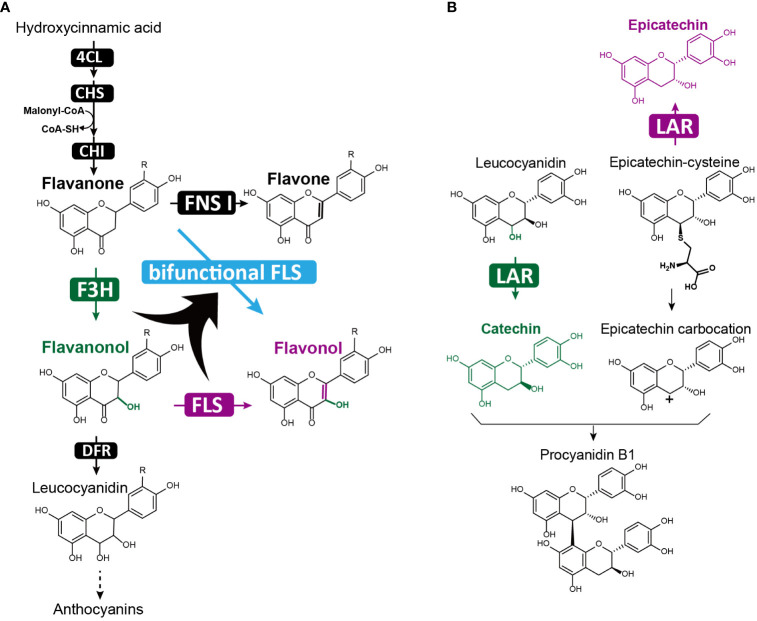
Bifunctional enzymes in flavonol and proanthocyanidin biosynthesis. **(A)** The bifunctional FLS enzyme (highlighted in blue) catalyzes two sequential reactions: adding a hydroxyl group at the C3 position and forming a double bond between the C2 and C3 positions of the flavonoid C-ring. In the canonical flavonoid biosynthetic pathway, these reactions are catalyzed separately by two single functional enzymes: F3H (highlighted in green) and FLS (highlighted in purple); **(B)** The bifunctional LAR performs dual functions in proanthocyanidin biosynthesis, converting leucoanthocyanidins to (+)-catechin (highlighted in green), and 4β-(*S*-cysteinyl)-epicatechin (epicatechin–cysteine) – a specialized form of the proanthocyanidin carbocation unit pool – into (-)-epicatechin (highlighted in purple).

Flavonols, a major flavonoid subgroup, are characterized by a hydroxyl group at the C3 position and a double bond between C2 and C3 positions of the C-ring. Known for their anti-inflammatory and antioxidant properties, flavonols have become important sources of drugs to treat diseases like cancer and trypanosomosis (Borsari et al., 2016; Salehi et al., 2020; Holland et al., 2023; Oo et al., 2022). Flavonol biosynthesis involves two critical modifications of the C-ring, namely C3 hydroxylation by F3H and C2-C3 bond desaturation by flavone synthase I (FNS I) or flavonol synthase (FLS). All three enzymes, FNS I, F3H, and FLS, belong to the 2-oxoglutarate-dependent dioxygenase (2-ODD) family (Farrow and Facchini, 2014), with FNS I and F3H exhibiting a closer phylogenetic relationship than FLS (Li et al., 2020). Bifunctional FLS enzymes have been identified in various plant species, including *Oryza sativa* (Park et al., 2019), *Brassica napus* (Schilbert et al., 2021), *Citrus unshiu* (Lukačin et al., 2003), *Ginkgo bilobaand* (Xu et al., 2012), and *Populus deltoides* (Kim et al., 2010), which can catalyze consecutive reactions of C3 hydroxylation and C2-C3 desaturation, producing flavonols from flavanones (**Figure 5A**). Although the evolutionary mechanisms leading to bifunctional FLS remain unclear, it is hypothesized that the dual-functional enzyme evolved early after plant terrestrialization, as promiscuous FNS I/F3H were observed in basal land plants such as liverworts and mosses (Li et al., 2020). From synthetic biology perspective, bifunctional FLS enzymes are useful for efficiently generating desired flavonols. Indeed, a study aiming to produce the plant flavonol kaempferol using yeast as a cell factory found that the bifunctional PdFLS or CitFLS alone yielded more kaempferol than co-expressing single-function enzymes MdFLS and ZmFLS (Duan et al., 2017).

Proanthocyanidins (PAs), or condensed tannins, are oligomeric flavonoids composed of flavan-3-ol units and can be found in numerous plants, particularly in berries such as cranberries, blueberries, and grape seeds. PAs and their oligomers serve as plant protectants against stress (Dixon et al., 2005), and are being investigated for their potential health benefits as therapeutics for cancer, cardiovascular diseases, and lipid metabolic disorders (Wang et al., 2020). The biosynthesis of PAs involves the polymerization of flavan-3-ol monomers, primarily (-)-epicatechin and (+)-catechin, with the flavan-3-ol carbocations attacking the C8 position of another monomer to extend the polymer (Dixon et al., 2005). Among the enzymes that generate flavan-3-ol monomers, leucoanthocyanidin reductase (LAR) play a crucial role in converting leucoanthocyanidins to (+)-catechin (Tanner et al., 2003) (**Figure 5B**). However, transgenic experiments have shown that LAR overexpression does not increase (+)-catechin production, but instead results in higher accumulation of (-)-epicatechin (Liu et al., 2013; Pang et al., 2013; Wang et al., 2018a). This led to the discovery of the second function of LAR, which converts 4β-(*S*-cysteinyl)-epicatechin(epicatechin–cysteine) – a special form of the PA carbocation unit pool – into (-)-epicatechin (Liu et al., 2016a; Yu et al., 2019) (**Figure 5B**). As a result, bifunctional LAR acts as a regulator of PA extension, fine-tuning the balance of insoluble long PA chains with soluble short PA chains and monomers (Liu et al., 2016a) (**Figure 5B**). Knocking out LAR in the legume *Medicago truncatula* disrupted this balance, cause an accumulation of insoluble PAs and a loss of soluble epicatechin-derived PAs (Liu et al., 2016a). In plant metabolic engineering, bifunctional LAR could be employed to manipulate the ratio of shorter and longer PA chains, as well as their biosynthetic monomeric precursors. This fine-tuning of PA composition by LAR provides an crucial approach for engineering health-promoting PAs in fruits, particularly optimizing PAs in grapevines, which directly affects red wine quality (Yu et al., 2019) (**Figure 5**).

### 5.3 Enzyme promiscuity and structure-guided glycosyltransferase engineering for flavonoid *C*-glycosylation

Flavonoids are commonly found as glycosides, including glucosides, glucuronsides, rhamnosides, and galactosides. The addition of sugar moieties to the flavonoid core significantly increases solubility and enhances bioavailability, influencing their pharmacological activity (Kren and Martinkova, 2001). Most glycosyl flavonoids are *O*-glycosylated with sugar moieties attached to hydroxyl groups. However, a survey of plant flavonoid chemical diversity revealed the presence of *C*-glycosides, especially in cereals (Brazier-Hicks et al., 2009). *C*-glycosylflavonoids are unique compounds with sugar groups directly conjugated to the aglycone via a C-C glycosidic bond. These flavonoids are highly stable against spontaneous and gastrointestinal hydrolysis due to the rigid C-C bond, resulting in a long *in vivo* half-life and remarkable druggability (Langenhan et al., 2005; Williams et al., 2007; Oualid and Silva, 2012).

Glycosyltransferases catalyze flavonoid glycosylation using activated sugar nucleotides as the glycosyl donor, such as UDP-glucose (UDP-Glc), UDP-galactose, and UDP-xylose (Slámová et al., 2018). However, compared to the relatively larger and well-studied *O*-glycosyltransferases (OGTs) family, only a few examples of *C*-glycosyltransferases (CGTs) have been reported in plants, such as *Oryza sativa* (Hao et al., 2016), *Glycine max* (Hirade et al., 2015), *Gentiana triflora* (Sasaki et al., 2015), *Fagopyrum esculentum* (Nagatomo et al., 2014), and *Pueraria lobata* (Wang et al., 2017b). The catalytic mechanism underlying *C*-glycosyl transfer remains unclear. However, recent comprehensive studies on promiscuous CGTs, capable of catalyzing *O*-, *C*-, and di-*C*-glycosylation, shed light on the mechanistic basis of CGT-mediated C-glycosylation (Gutmann and Nidetzky, 2012; Chen et al., 2018; He et al., 2019; Zhang et al., 2020; Dai et al., 2021). These findings have paved the way for developing efficient and versatile CGTs through protein engineering.

**Figure 6:**
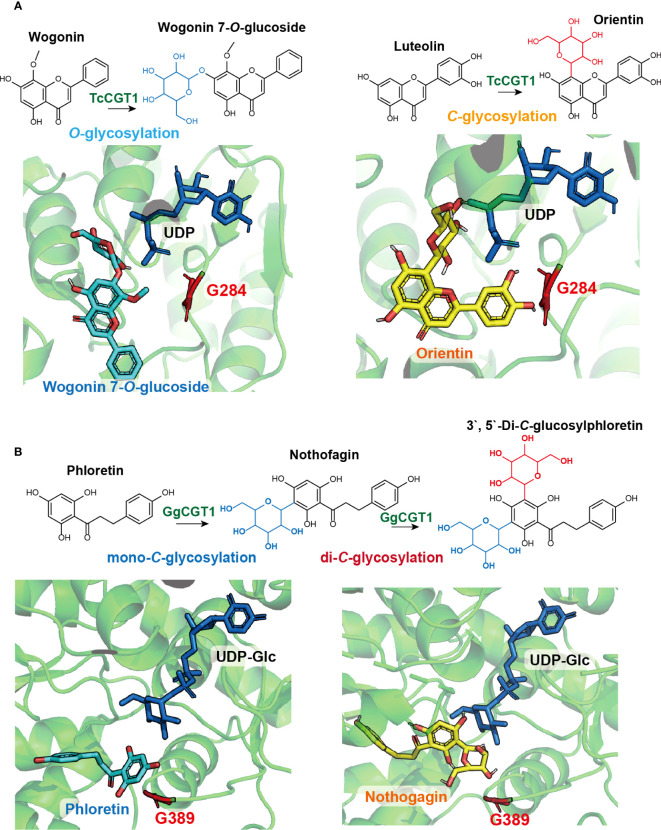
Natural variation and structure-function-guided exploration of key amino acid residues affecting flavonoid *C*-glycosylation. **(A)** TcCGT1 from Trollius chinensis is a promiscuous enzyme catalyzing both *C*- and *O*-glycosylation using apigenin and wogonin as acceptor substrates, respectively. Protein structural analysis and molecular docking reveal the binding pose of the *C*-glycosylation product, wogonin 7-*O*-glucoside (blue), is nearly perpendicular to the *C*-glycosylation product, orientin. The key residue G284 (red) allows for a more spacious acceptor binding pocket for the *C*-glycosyl acceptor; **(B)** GgCGT from Glycyrrhiza glabra can perform consecutive *C*-glycosylation to produce di-*C*-glycosylflavonoid, 3’,5’-Di-*C*-glucosylphloretin. Protein structural analysis and molecular docking suggested that after the first round of *C*-glycosylation, the acceptor substrate, phloretin (blue), may rotate in the binding pocket, exposing the second *C*-glycosylation site to UDP-Glc for the subsequent glycosyl transfer. The key residue G389 (red) provides a spacious pocket to accommodate nothofagin (yellow), the mono-*C*-glycosylation product from the first round of glycosylation.

Recent studies have identified key amino acid residues in glycosyltransferases (GTs) that contribute to the switch between *O*- and *C*-glycosylation and have applied them for enzyme engineering (Gutmann and Nidetzky, 2012; He et al., 2019). For instance, a notably promiscuous TcCGT1 from *Trollius chinensis* was found to catalyze C-glycosylation or *O*-glycosylation using 41 out of 114 tested structurally diverse flavonoid substrates with UDP-Glc as the sugar donor(He et al., 2019). Using apigenin and wogonin as the respective *C*- and *O*-glycosylation acceptors (**Figure 6**), protein structural analysis coupled with molecular docking revealed that the binding pose of the C-glycosyl acceptor is almost vertical to the *O*-glycosyl acceptor (He et al., 2019), thus necessitating a more spacious acceptor binding pocket, partially determined by the key residue G284. Mutating G284 to bulkier residues such as Phe, Gln, Tyr, or Lys significantly reduced C-glycosylation activity of TcCGT1. Introducing a salt-bridge between G284 and I94 through site-directed mutagenesis of G284F, G284Q, G284Y, and I94E/G284K completely blocked C-glycosylation and turned TcCGT1 into a single functional OGT (He et al., 2019). In another study, Gutmann and Nidetzky identified the active motif in the single-functional enzymes OsCGT and PcOGT using structural-functional analysis (Gutmann and Nidetzky, 2012). They found that reciprocally switching the key residues in the active motif (I117 and D118) in PcOGT to (D120 and I121) in OsCGT created promiscuous enzymes capable of catalyzing both *O*- and *C*-glycosylation with the dihydrochalcone phloretin as the acceptor (Gutmann and Nidetzky, 2012).

Among the diverse plant CGTs, some are capable of performing consecutive *C*-glycosylation to produce di-*C*-glycosylflavonoids (Chen et al., 2018; Zhang et al., 2020; Dai et al., 2021). Zhang et al. identified a promiscuous GgCGT from *Glycyrrhiza glabra*, which could catalyze the *C*-glycosylation of 33 out of 51 tested phenolic compounds as the acceptor substrates, with six compounds converted to di-*C*-glucosides (Zhang et al., 2020). The crystal structure of GgCGT was solved using the proteins in complex with sugar donor UDP-Glc and acceptor phloretin. The binding poses of UDP-Glc, phloretin, and its mono-*C*-glycoside nothofagin in the enzyme reaction center were investigated (Zhang et al., 2020). This analysis revealed that the acceptor phloretin possibly rotates in the binding pocket after the first round of *C*-glycosylation, exposing the second site of C-glycosylation to UDP-Glc for the glycosyl transfer reaction. The binding pose of nothofagin requires a spacious pocket that is determined by the key residue G389. Replacing G389 with bulkier residues Arg or Trp hindered the enzymatic reaction of the second round of *C*-glycosylation, whereas mutating G389 into Lys almost completely turned the enzyme into a mono-C-glycosyltransferase (Zhang et al., 2020). In a similar study, enzyme promiscuity was leveraged to examine two recently diverged CGTs from *Mangifera indica* and to uncover key residues contributing to di-*C*-glycosylation (Chen et al., 2018). Although MiCGT and MiCGTb share 90% amino acid sequence similarity, MiCGTb could transfer two *C*-glycosyl groups to phloretin, while MiCGT only catalyzes mono-*C*-glycosylation. A single amino acid – either I152 or E152 – located in the enzyme binding pocket was pinpointed as influencing donor-acceptor substrate permissiveness and determining the transition between mono-*C* and di-*C*-glycosylation (Chen et al., 2018). These catalytic residues that switch flavonoid *O*-, mono-*C*-, or di-*C*-glycosylation further expand the toolbox for metabolic engineering of structurally diverse and pharmacologically active flavonoid *C*-glycosides. The identification of key amino acid residues of GTs contributing to the switch between *O*- and *C*-glycosylation, such as those identified in GgCGT and *Mangifera indica* CGTs, can be applied for enzyme engineering in the production of valuable *C*-glycosides.

## Conclusion

Recent advancements in plant synthetic biology have demonstrated the significant potential of utilizing plants as alternative hosts for metabolic engineering. This approach offers a promising platform to produce plant-derived nutrients or bioactive chemicals that are difficult to produce in microbes but can be more easily manipulated in plants by modifying existing regulatory or biosynthetic enzymes. In particular, flavonoid metabolic engineering in plants has been successfully pursued in two main directions: boosting flavonoid content by manipulating transcription regulatory components and introducing novel flavonoids through engineering enzyme diversity discovered by exploring natural plant variation. By leveraging these two strategies, future plant flavonoid metabolic engineering will provide a variety of approaches to alter the flavor, aroma, and color of plant-based products, making them more appealing to consumers and benefiting the food, beverage, and pharmaceutical industries.

## Author contributions

PF, LJ, YG, WZ, and LH contributed to the conceptualization of the review. LJ and PF wrote the first draft of the manuscript. YG, WZ, and LH reviewed and edited the manuscript. All authors contributed to the article and approved the submitted version.
